# Supplementary material for: In Vitro Newly Isolated Environmental Phage Activity against Biofilms Preformed by Pseudomonas aeruginosa from Patients with Cystic Fibrosis
Source: Microorganisms. 2021 Feb 25;9(3):478. doi: 10.3390/microorganisms9030478 (PMC7996588; doi:10.3390/microorganisms9030478)
Supplement: Supplementary file 1 [file microorganisms-09-00478-s001.pdf]

microorganisms

Supplementary Materials: In vitro Newly Isolated Environmental Phage Activity against Biofilms Preformed by Pseudomonas aeruginosa from Patients with Cystic Fibrosis

Ersilia Vita Fiscarelli [1,†], Martina Rossitto [1,†], Paola Rosati [2,*], Nour Essa [1], Valentina Crocetta [3], Andrea Di Giulio [4], Veronica Lupetti [3], Giovanni Di Bonaventura [3,‡] and Arianna Pompilio [3,‡]

Table S1. The 22 newly isolated bacteriophages (phages) from five environmental sewage sources near Rome and in Rome, including Bambino Gesù Children's Hospital (OBG), Italy. Characteristics, titers, and phage cross-activity vs. indicator bacteria (laboratory *Pseudomonas aeruginosa*, PAO1, and six PA isolates from patients with cystic fibrosis, CF, namely MA1-MA6).

Phages	Sewage Sources (Five Sources including OBG)	Primary PA Hosts (Laboratory PAO1 and CF PA MA4)	Phage Plaque Morphology			Phage Titers (PFU/mL) [a]	Indicator Bacteria (Laboratory PAO1 and Six CF PA MA1-MA6) Tested for Phage Cross-Activity
			Size (mm) [b]	Aspect [c]	Halo [d]		
Φ1_DL1	Rome	MA4	3	C	+	1.0×10^6	MA1, MA3, PAO1
Φ2_DL1	Rome	MA4	2	C	+	2.0×10^6	MA1, MA3, PAO1
Φ3_ZP1	Farm-house 1	MA4	2	C	+	3.0×10^6	MA1, MA3, PAO1
Φ4_ZP1	Farm-house 1	MA4	3	T	+	5.0×10^7	MA1, MA3, MA5, PAO1
Φ5_ZP1	Farm-house 1	MA4	1	C	+	1.0×10^8	MA1, MA3, PAO1
Φ6_ZP2	Farm-house 2	MA4	3	C	+	2.0×10^4	MA1, MA3, PAO1
Φ7_ZP2	Farm-house 2	MA4	3	C	+	1.2×10^6	MA1, MA3, PAO1
Φ8_ZP2	Farm-house 2	MA4	2	C	+	1.5×10^7	MA1, MA3, PAO1
Φ9_ZP2	Farm-house 2	MA4	1	C	+	6.0×10^6	MA1, MA3, MA6, PAO1
Φ10_ZP2	Farm-house 2	MA4	3	C	+	3.0×10^5	MA1, MA3, PAO1
Φ11_ZP3	Farm-house 3	MA4	2	C	+	1.3×10^4	MA1, MA3, PAO1
Φ12_ZP3	Farm-house 3	MA4	3	C	+	1.4×10^7	MA1, MA3, PAO1

Φ13_OBG	OBG[g]	MA4	3	C	+	1.1×10^{6}	MA1, MA3, PAO1
Φ14_OBG	OBG	MA4	2	C	+	1.0×10^{7}	MA1, MA3, PAO1
Φ15_OBG	OBG	MA4	1	C	+	2.0×10^{8}	MA1, MA3, PAO1
Φ16_OBG	OBG	PAO1	5	T	-	6.0×10^{7}	MA1, MA2, MA3, MA4, MA5
Φ17_OBG	OBG	PAO1	4	C	-	1.3×10^{9}	MA1, MA2, MA3, MA4, MA5
Φ18_OBG	OBG	PAO1	3	C	-	1.0×10^{10}	MA1, MA2, MA3, MA4, MA5
Φ19_OBG	OBG	PAO1	2	C	-	1.1×10^{9}	MA1, MA2, MA3, MA4, MA5
Φ20_OBG	OBG	PAO1	4	C	-	1.9×10^{9}	MA1, MA2, MA3, MA4, MA5
Φ21_OBG	OBG	PAO1	2	C	-	1.0×10^{8}	MA1, MA2, MA3, MA4, MA5
Φ22_OBG	OBG	PAO1	2	C	-	2.0×10^{8}	MA1, MA2, MA3, MA4, MA5

[a] PFU/mL = plaque forming units. [b] mm = millimiters. [c] Clear, C; Turbid, T. [d] Absence, - ; presence, +.

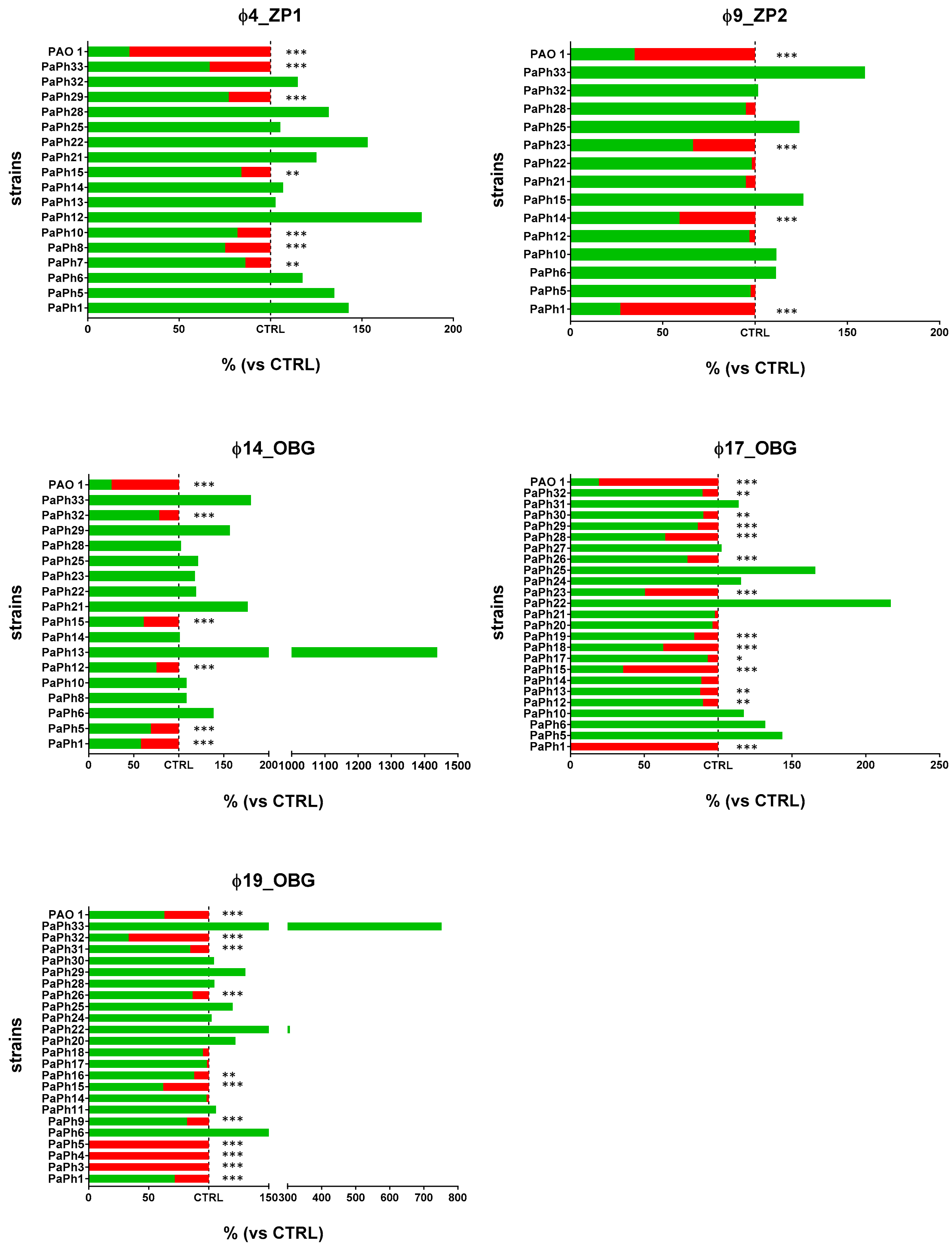

Figure S1. *Pseudomonas aeruginosa* (PA) biofilm dispersions evaluated i*n vitro* after being exposed for 4-h to the five selected newly isolated environmental phages (Φ4_ZP1, Φ9_ZP2, Φ14_OBG, Φ17_OBG and Φ19_OBG) tested against laboratory PA (PAO1) and 33 cystic fibrosis PA 24-h-old biofilms at multiplicity of infection (MOI) 1. Biofilm dispersions evaluated by spectrophotometry in crystal violet stain. Results are shown as percentages (%) dispersed PA biofilms (red) and residual biofilm biomasses (green) when comparing phage exposed and unexposed PA biofilms treated with trypticase soy broth (TSB) control samples (CTRL). The dotted line represents 100% residual biofilms in controls after TSB challenge. The green lines over the dotted line represent PA biofilm biomasses increase when comparing phage exposed and unexposed CTRL PA biofilms. Significant levels * $p < 0.05$, ** $p < 0.01$, *** $p < 0.001$ analyzed by the χ^2test.

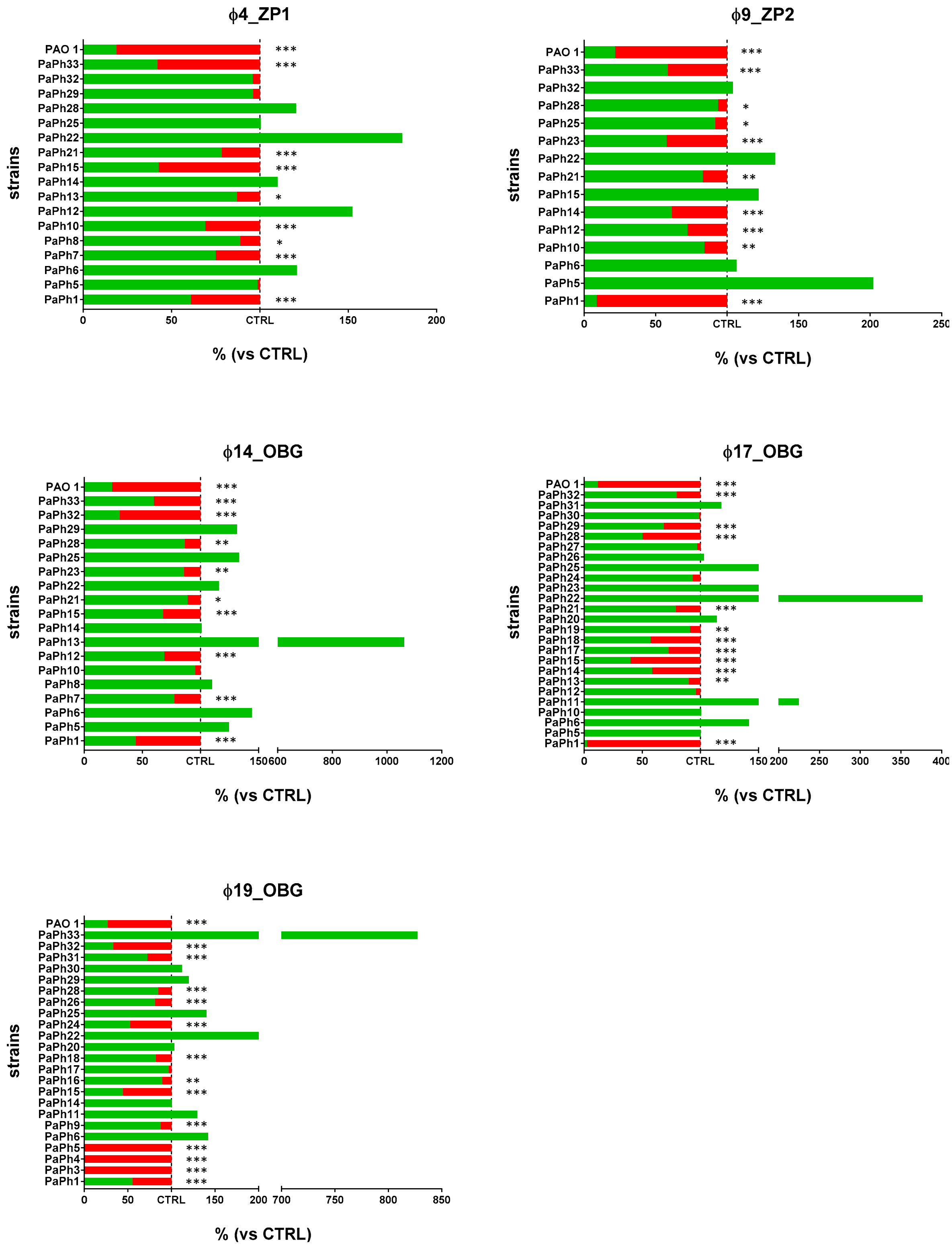

Figure S2. *Pseudomonas aeruginosa* (PA) biofilm dispersions evaluated *in vitro* after exposure for 4-h to the five selected newly isolated environmental phages (Φ4_ZP1, Φ9_ZP2, Φ14_OBG, Φ17_OBG and Φ19_OBG) tested against laboratory PA (PAO1) and 33 cystic fibrosis PA 24-h-old biofilms at multiplicity of infection (MOI) 100. Biofilm dispersions evaluated by spectrophotometry in crystal violet stain. Results are shown as percentages (%) dispersed PA biofilms (red) and residual biofilm biomasses (green) when comparing phage exposed and unexposed PA biofilms treated with trypticase soy broth (TSB) control samples (CTRL). The dotted line represents 100% residual biofilms in controls after TSB challenge. The green lines over the dotted line represent PA biofilm biomasses increase when comparing phage exposed and unexposed CTRL PA biofilms. Significant levels * p <0.05, ** p < 0.01, *** p <0.001 analyzed by the χ^2 test.

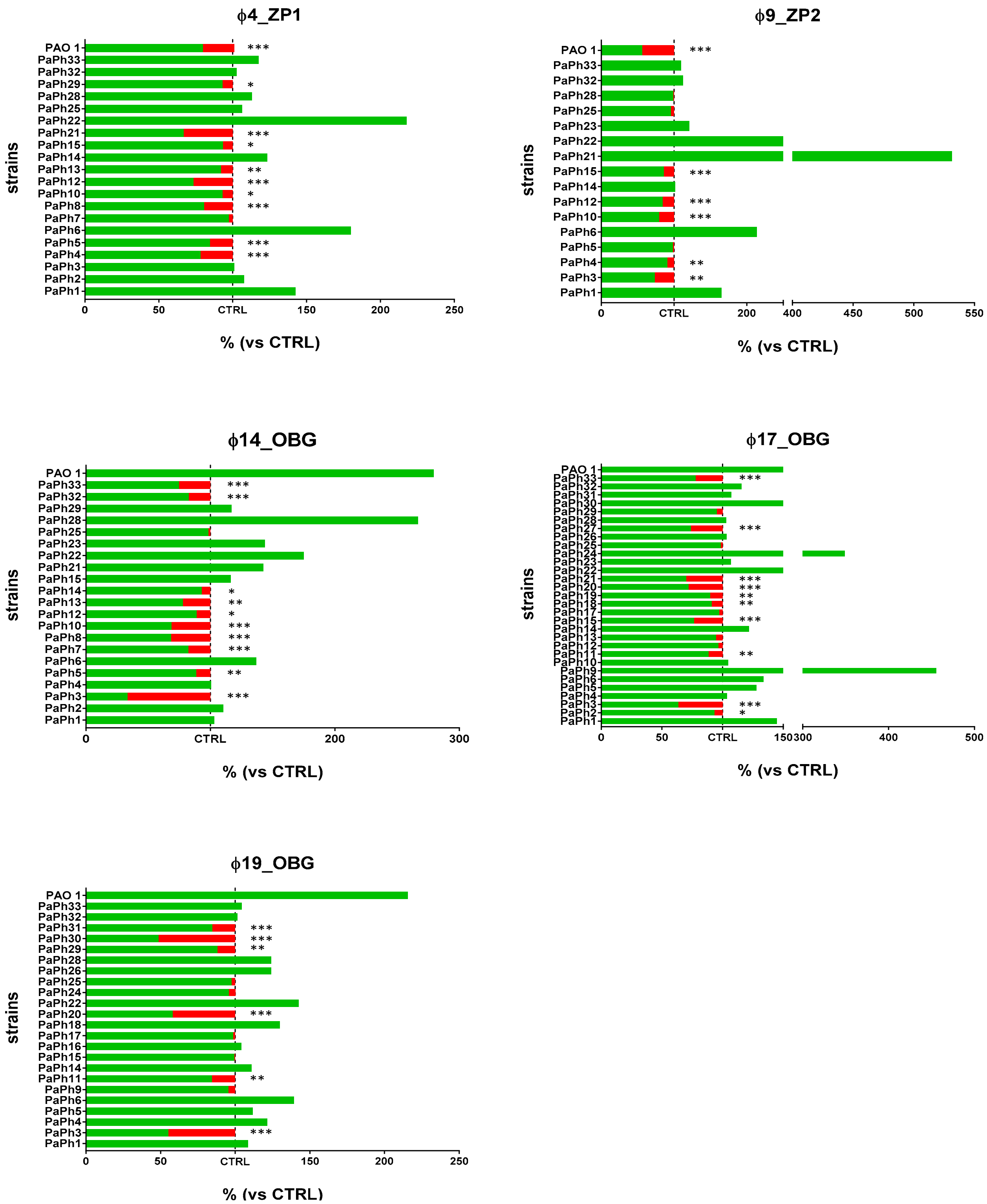

Figure S3. *Pseudomonas aeruginosa* (PA) biofilm dispersions evaluated *in vitro* after 24-h exposures to the five newly isolated environmental selected phages (Φ4_ZP1, Φ9_ZP2, Φ14_OBG, Φ17_OBG and Φ19_OBG) tested against laboratory PA (PAO1) and 33 cystic fibrosis PA 24-h-old biofilms at multiplicity of infection (MOI) 1. Biofilm dispersions evaluated by spectrophotometry in crystal violet stain. Results are shown as percentages (%) dispersed PA biofilms (red) and residual biofilm biomasses (green) when comparing phage exposed and unexposed PA biofilms treated with trypticase soy broth (TSB) control samples (CTRL). The dotted line represents 100% residual biofilms in controls after TSB challenge. The green lines over the dotted line represent PA biofilm biomasses increase when comparing phage exposed and unexposed CTRL PA biofilms. Significant levels * $p < 0.05$, ** $p < 0.01$, *** $p < 0.001$ analyzed by the χ^2 test.

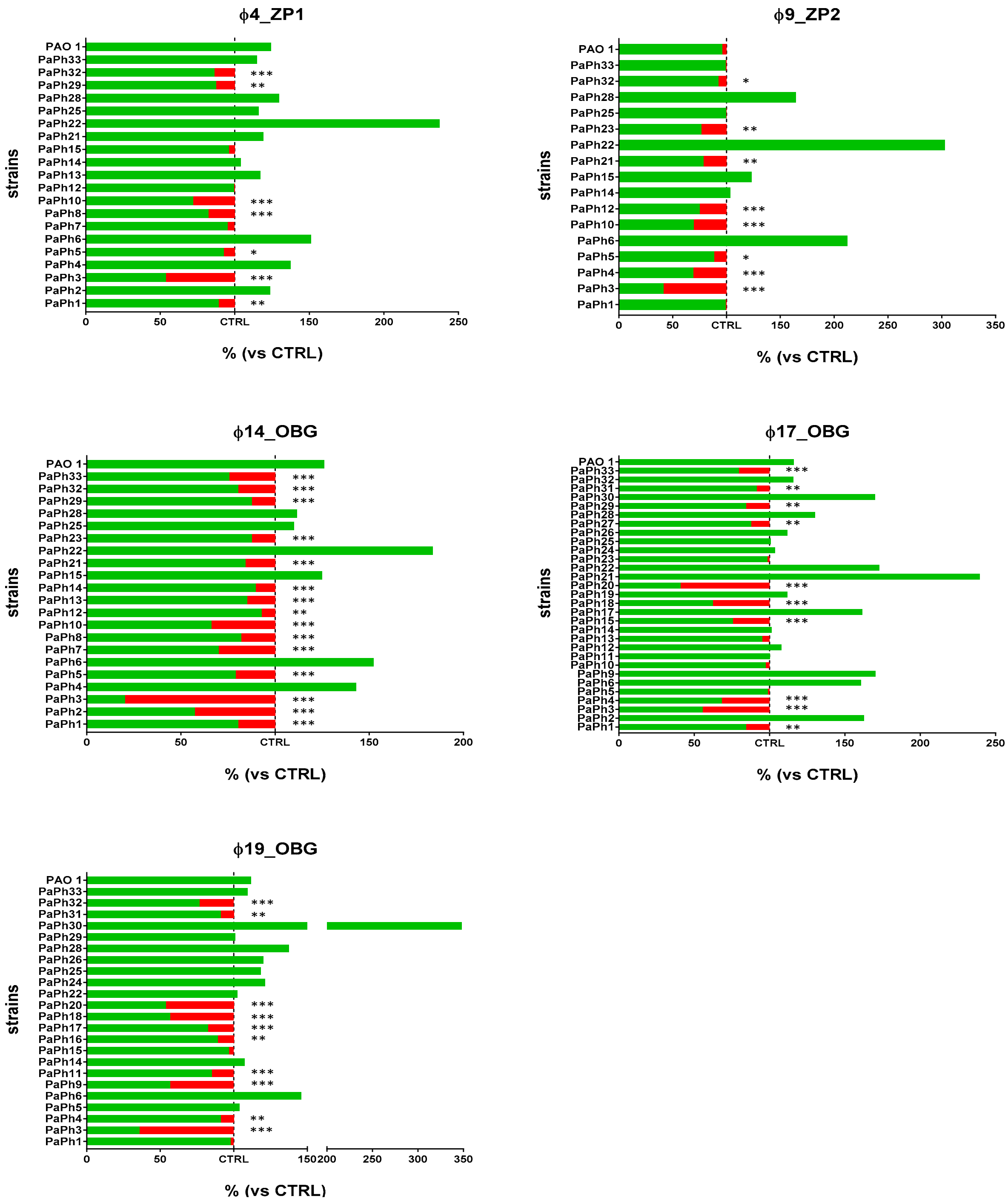

Figure S4. *Pseudomonas aeruginosa* (PA) biofilm dispersions evaluated i*n vitro* after 24-h exposures to the five newly isolated environmental selected phages (Φ4_ZP1, Φ9_ZP2, Φ14_OBG, Φ17_OBG and Φ19_OBG) tested against laboratory PA (PAO1) and 33 cystic fibrosis PA 24-h-old biofilms at multiplicity of infection (MOI) 100. Biofilm dispersions evaluated by spectrophotometry in crystal violet stain. Results are shown as percentages (%) dispersed PA biofilms (red) and residual biofilm biomasses (green) when comparing phage exposed and unexposed PA biofilms treated with trypticase soy broth (TSB) control samples (CTRL). The dotted line represents 100% residual biofilms in controls after TSB challenge. The green lines over the dotted line represent PA biofilm biomasses increase when comparing phage exposed and unexposed CTRL PA biofilms. Significant levels * $p < 0.05$, ** $p < 0.01$, *** $p < 0.001$ analyzed by the χ^2 test.
